# Permissible noninformative priors for the accelerated life test model with censored data

**DOI:** 10.1186/s40064-016-2004-0

**Published:** 2016-03-24

**Authors:** Fode Zhang, Yimin Shi

**Affiliations:** Department of Applied Mathematics, Northwestern Polytechnical University, Xi’an, 710072 Shaanxi People’s Republic of China

**Keywords:** Jeffreys priors, Permissible priors, Partially accelerated life test, Progressive Type-II censoring, Metroplis with in Gibbs sampling, 62N01, 62N05, 62C10, 62F15

## Abstract

In this paper, the Jeffreys priors for the step-stress partially accelerated life test with Type-II adaptive progressive hybrid censoring scheme data are considered. Given a density function family satisfied certain regularity conditions, the Fisher information matrix and Jeffreys priors are obtained. Taking the Weibull distribution as an example, the Jeffreys priors, posterior analysis and its permissibility are discussed. The results, which present that how the accelerated stress levels, censored size, hybrid censoring time and stress change time etc. affect the Jeffreys priors, are obtained. In addition, a theorem which shows there exists a relationship between single observation and multi observations for permissible priors is proved. Finally, using Metroplis with in Gibbs sampling algorithm, these factors are confirmed by computing the frequentist coverage probabilities.

## Background

Noninformative priors, which make the Bayesian analysis is a distinct field in some sense, have received a lot of attention in the past decades (see references Berger et al. 2009, 2014; Guan et al. 2013; Jeffreys 1946). Among important noninformative priors are Jeffreys priors because of invariant and good frequentist properties.

Given a model $\{p(y|{\theta }), y\in {\mathcal {Y}},{\theta }\in \Theta \}$ and a prior $\pi ({\theta })$, for any invertible transform $\eta =\eta ({\theta })$, the prior $\pi ({\theta })$ is said to be satisfied Jeffreys’ rule if

1 $$\begin{aligned} \pi ({\theta })=\pi (\eta )|J(\eta )|, \end{aligned}$$

where $|J(\eta )|$ is the Jacobian of the transformation from $\eta$ to ${\theta }$.

Jeffreys (1946) proposed the famous Jeffreys prior

2 $$\begin{aligned} \pi ({\theta })=\sqrt{|I({\theta })|} \end{aligned}$$

satisfies the Jeffreys’ rule (1), where $|I({\theta })|$ denotes the determinant of the expected Fisher information matrix $I({\theta })$. The another important property of Jeffreys prior is its good frequentist properties.

For a prior $\pi ({\theta })$, given a set of observations $\mathbf y$ of size *n*. Let ${\theta }_{n}^{\pi }({\alpha }|\mathbf y )$ denote the ${\alpha }$th quantile of the posterior distribution of ${\theta }$, i.e.,

$$\begin{aligned} P\left( {\theta }\le {\theta }_{n}^{\pi }({\alpha }|{\mathbf y} )|{\mathbf y} \right) ={\alpha }. \end{aligned}$$

A prior $\pi$ is said to be an *i*th order matching prior for ${\theta }$ if

$$\begin{aligned} P\left( {\theta }\le {\theta }_{n}^{\pi }({\alpha }|{\mathbf y} )|{\theta }\right) ={\alpha }+O(n^{-\frac{i}{2}}). \end{aligned}$$

In the uniparametric case, for any smooth prior $\pi ^{*}({\theta })$ under regularity conditions, Welch and Peers (1963) show that the frequentist coverage probability is

$$\begin{aligned} P\left( {\theta }\le {\theta }_{n}^{\pi }({\alpha }|{\mathbf y} )|{\theta }\right) ={\alpha }+O(n^{-\frac{1}{2}}). \end{aligned}$$

That is $\pi ^{*}({\theta })$ is the first-order matching prior. However, using the Jeffreys prior $\pi ({\theta })$, then

$$\begin{aligned} P\left( {\theta }\le {\theta }_{n}^{\pi }({\alpha }|{\mathbf y} )|{\theta }\right) ={\alpha }+O(n^{-1}), \end{aligned}$$

that is Jeffreys prior $\pi ({\theta })$ is the second-order matching prior. Moreover, Welch and Peers (1963) also show that Jeffreys prior is the unique second-order matching prior under certain regularity conditions.

In the survival analysis, there are exist two most popularly methods to save the experiment cost, one is accelerated life test, the another is censored data. As products become highly reliable with substantially long life-spans, time-consuming and expensive tests are often required to collect a sufficient amount of failure data for analysis. This problem has been solved by use of accelerated life tests (ALT), in which the units are subjected to higher than normal stress levels, like pressure, voltage, vibration and temperature, etc., to induce rapid failures. The test is said to be SSPALT (see references Dharmadhikari and Rahman 2003; Han 2015; Ismail 2014; Wu et al. 2014; Abd-Elfattah et al. 2008), if a test unit is first run at normal condition and, if it does not fail for a specified time, then it is run at accelerated stress until failure occurs or the observation is censored.

In reliability experiments, the another way to to save time and reduce cost is censored data (see references Voltermana et al. 2014; Wu et al. 2014; Balakrishnan and Kundu 2013; Park et al. 2015. If the experimental time is fixed, we called the scheme is Type-I censoring scheme, in this case the number of observed failures is a random variable. Otherwise, if the number of observed failures is fixed, we called the scheme is Type-II censoring scheme, in this case the experimental time is a random variable.

In this paper, we investigate the Jeffreys priors for SSPALT with Type-II APHCS, this censoring scheme will be illustrated in the next section. The main results of this paper can be briefly described as follows.

- Given a model $\{p(y|{\theta }),y\in {\mathcal {Y}},{\theta }\in \Theta \}$ under certain regularity conditions, the likelihood function for the SSPALT with Type-II APHCS data is unified, and is done in “Likelihood function and special sub-likelihood functions” section.
- The elements of Fisher information matrix are investigated under different censoring schemes. The relationships among with the Jeffreys priors which obtained from censored data and uncensored data are researched, and are done in “Jeffreys priors for survival models” section.
- Taking the Weibull distribution as an example, the Jeffreys priors $$\pi _{1J}({\theta }),$$$$\pi _{2J}({\theta }),$$$$\pi _{3J}({\theta })$$ and $$\pi _{4J}({\theta })$$ based on the SSPALT with Type-II APHCS, Type-II APHCS, Type-II CS and complete samples are discussed, respectively, and are done in “Jeffreys priors for the Weibull distribution” section.
- The posterior analyses based on the Jeffreys priors are studied in “Posterior analysis” section.
- The permissibility of Jeffreys priors is analyzed, and a theorem which shows that there exists a relationship between single observation and multi observations for permissible prior is proved, and are done in “Permissible Jeffreys priors” section.
- Using the random way Metroplis with in Gibbs sampling techniques, simulation is acquired in “Simulation studies and frequency analysis” section.

For convenience, we define the following notations:

$$n_{u}$$:
the number of observed items at normal condition.
$$n_{a}$$:
the number of observed items at accelerated condition.
$$c_{u}$$:
times of censored items at normal condition.
$$c_{a}$$:
times of censored items at accelerated condition.
*k*: acceleration factor.
$$\tau$$:
stress change time.
$$\eta$$:
hybrid censoring time.
$$I_{A}$$:
indicator function on a set *A*.
$$R_{i}$$:
the number of units removed at the time of the *i*th failure.
$${\mathbf y} ^{n}$$:
vector of $(y_{1},\dots ,y_{n})$, specially ${\mathbf y} ^{0}=0$.
$${\mathbf y} ^{i,j}$$:
vector of $(y_{i},\dots ,y_{j})$ for $1< i< j\le n$, 0 for $i>j$, $y_{i}$ for $i=j$.
$$y_{i:m:n}$$:
*i*th observed failure times.
$$A\mathop {=}\limits ^{\mathrm {def}}B$$:
B can be defined by A.

## Progressive hybrid Type-II censoring schemes

In order to overcome the drawbacks of Type-I and Type-II censoring schemes, the mixture of Type-I and Type-II censoring schemes, which known as hybrid censoring scheme, was originally introduced by Epstein (1954). If each of the failure times, there are some surviving units are randomly removed from the experiment, and all the remaining surviving units are removed from the experiment at the time when the conditions of the terminate experiment are satisfied, we said this scheme is progressive censoring scheme (see Balakrishnan and Aggarwala 2000; Balakrishnan and Kundu 2013). In this paper, we consider the Type-II APHCS (see Ng et al. 2009) which can be defined as follows:

1.: Suppose *n* items are placed on a life-test, the effective sample size $m<n$ and time $\eta$ are fixed in advance.
2.: At the time of the first failure denoted $Y_{1:m:n}$, $R_{1}$ of the remaining $n-1$ surviving units are randomly removed from the experiment.
3.: The experimental continues, at the time of the *k*th failure denoted $Y_{k:m:n}$, $R_{k}$ of the remaining $n-k-\sum _{i=1}^{k-1}R_{i}$ surviving units are randomly removed from the experiment.
4*a*.: If the *m*th failure time occurs before time $$\eta$$$$(i.e., Y_{m:m:n}<\eta )$$, the experiment will be terminated at time $$Y_{m:m:n}$$ and all remaining $$n-m-\sum _{i=1}^{m-1}R_{i}$$ surviving units are removed from the experiment.
4*b*.: Otherwise, once the experimental time passes time $\eta$, but the number of observed failures has not yet reached *m*$(i.e., Y_{j:m:n}<\eta <Y_{j+1:m:n}<Y_{m:m:n})$. They do not withdraw any units at all except for the time of the *m*th failure where all remaining $n-m-\sum _{i=1}^{j}R_{j}$ surviving items are removed.

From now on, we use the censoring scheme *a* and the censoring scheme *b* denote the schemes $(1){\rightarrow }(2){\rightarrow }(3){\rightarrow }(4a)$ and $(1){\rightarrow }(2){\rightarrow }(3){\rightarrow }(4b)$, respectively.

## Likelihood function and special sub-likelihood functions

For simplicity, let ${\mathbf Y} ^{m}=(Y_{1},Y_{2},\dots ,Y_{m})\mathop {=}\limits ^{\mathrm {def}}(Y_{1:m:n},Y_{2:m:n},\dots ,Y_{m:m:n})$ denotes a Type-II APHCS sample from a density function family $\{f(y|{\theta }),y\in {\mathcal {Y}},{\theta }\in \Theta \}$ under Cramer–Rao regularity conditions, with distribution function $F(y)\mathop {=}\limits ^{\mathrm {def}}F(y\mid {\theta })$ and survival function $S(y)\mathop {=}\limits ^{\mathrm {def}}S(y\mid {\theta })$.

Based on the transformation variable technique proposed by DeGroot and Goel (1979):

$$\begin{aligned} Y=\left\{ \begin{array}{lll} T, &{}\quad if \;T\le \tau , \\ \tau +(T-\tau )/k,&{}\quad if \;T\ge \tau ,\\ \end{array} \right. \end{aligned}$$

where *T* is the lifetime of the unit under normal use condition, $\tau$ is the stress change time and $k>1$ is the acceleration factor. The density function and survival function of *Y* under SSPALT model can be given by, respectively,

$$\begin{aligned} f(y)& = {} \left\{ \begin{array}{lll} 0, &{}\quad y\le 0, \\ f_{1}(y)=f(y)\mathop {=}\limits ^{\mathrm {def}}f(y\mid {\theta }),&{}\quad 0<y\le \tau ,\\ f_{2}(y),&{}\quad \tau <y,\\ \end{array} \right. \\ S(y)& = {} \left\{ \begin{array}{lll} 0, &{}\quad y\le 0, \\ S_{1}(y)=S(y)\mathop {=}\limits ^{\mathrm {def}}S(y\mid {\theta }),&{}\quad 0<y\le \tau ,\\ S_{2}(y),&{}\quad \tau <y,\\ \end{array} \right. \end{aligned}$$

where

$$\begin{aligned} f_{2}(y)\mathop {=}\limits ^{\mathrm {def}}k f(\tau +k(y-\tau )\mid {\theta }), \,\,S_{2}(y)\mathop {=}\limits ^{\mathrm {def}}k S(\tau +k(y-\tau )\mid {\theta }). \end{aligned}$$

We suppose, without loss of generality, that $\tau \le \eta$ except for particular pointed place. In order to unify the likelihood function, we introduce the following indicator functions:

$$\begin{aligned} {\delta }_{1i}=I_{Y_{i}\le \tau }, \,\,\,\,\overline{{\delta }}_{1i}=I_{\tau <Y_{i}\le Y_{m}},\,\,\, {\delta }_{2i}^{c\eta }=I_{Y_{n_{u}+1}\le Y_{i}\le Y_{j}\cup Y_{i}=Y_{m}}. \end{aligned}$$

Then, based on the Ismail (2014), the joint density function of SSPALT model with Type-II APHCS data is given by

3 $$\begin{aligned} L({\theta };{\mathbf y} ^{m})=\prod _{i=1}^{m}f_{1}^{{\delta }_{1i}}(y_{i})S_{1}^{R_{i}{\delta }_{1i}}(y_{i}) f_{2}^{\overline{{\delta }}_{1i}}(y_{i})S_{2}^{R_{i}{\delta }_{2i}^{c}}(y_{i}), \end{aligned}$$

where

$$\begin{aligned} {\delta }_{2i}^{c}=\left\{ \begin{array}{lll} {\delta }_{2i}^{c\eta }, &{}\quad Y_{j}<\eta <Y_{j+1}\le Y_{m}, \\ \overline{{\delta }}_{1i},&{}\quad Y_{m}<\eta ,\\ \end{array} \right. \end{aligned}$$

$R_{m}=n-m-\sum _{i=1}^{j}R_{i}$, for $Y_{j}<\eta <Y_{j+1}\le Y_{m}$, and $R_{m}=n-m-\sum _{i=1}^{m-1}R_{i}$, for $Y_{m}<\eta .$ Obviously

$$\begin{aligned} \sum _{i=1}^{m}{\delta }_{1i}=n_{u}=c_{u},\quad \sum _{i=1}^{m}\overline{{\delta }}_{1i}=n_{a},\quad \sum _{i=1}^{m}{\delta }_{2i}^{c}=c_{a}. \end{aligned}$$

Assume $D_{1}$ and $C_{1}$ are the sets of individuals for whom lifetimes are observed and censored at normal conditions, respectively. Similarly, suppose $D_{2}$ and $C_{2}$ are the sets of individuals for whom lifetimes are observed and censored at accelerated conditions, respectively. Then the likelihood function (3) can be rewritten as

4 $$\begin{aligned} L({\theta };{\mathbf y} ^{m})=\prod _{i\in D_{k}}f_{k}(y_{i}\mid {\theta })\prod _{i\in C_{k}}S_{k}^{R_{i}}(y_{i}\mid {\theta }),\quad k=1,2. \end{aligned}$$

### *Remark 1*

Consider some special cases, we have the following results.

- If ${\mathbf R} ^{m-1}={\mathbf 0}$, $\tau <y_{m}$, then the Eq. (4) can be reduced to
  5 $$\begin{aligned} L({\theta };{\mathbf y} ^{m})=\prod _{i\in D_{k}}f_{k}(y_{i}\mid {\theta })S_{2}^{R_{m}}(y_{m}\mid {\theta }),\quad k=1,2, \end{aligned}$$
  that is the likelihood function for a SSPALT model with Type-II censored data.
- Let the stress change time $\tau$ be big enough, i.e., $\tau >y_{m}$. Then, the accelerated stress is invalid in the life test, the Eq. (4) becomes
  6 $$\begin{aligned} L({\theta };{\mathbf y} ^{m})=\prod _{i\in D_{1}}f_{1}(y_{i}\mid {\theta })\prod _{i\in C_{1}}S_{1}^{R_{i}}(y_{i}\mid {\theta }), \end{aligned}$$
  that is the likelihood function for a life test model with Type-II APHCS data.
- Let the pre-specified time $\eta$ and stress change time $\tau$ are big enough such that $y_{m}<\min \{\tau ,\eta \}$, then the Eq. (4) can be simplified to
  7 $$\begin{aligned} L({\theta };{\mathbf y} ^{m})=\prod _{i=1}^{m}f_{1}(y_{i}\mid {\theta })S_{1}^{n-m}(y_{m}\mid {\theta }), \end{aligned}$$
  that is the likelihood function for a life test model with Type-II censored data.

## Jeffreys priors for survival models

In fact, much work has been done to study the Jeffreys priors for life test with censored data. This goes to the early works of Santis et al. (2001) and Fu et al. (2012).

Let ${\mathbf Y} ^{n}$ be a set of data with size *n*, $L({\theta };{\mathbf y} ^{n})$ be a likelihood function for an unknown parameter ${\theta }$, then the Jeffreys prior for ${\theta }$ is defined by

$$\begin{aligned} \pi _{{\theta }}^{J}=\sqrt{\mid I({\theta })\mid }, \end{aligned}$$

where $\mid I({\theta })\mid$ denotes the determinant of the expected Fisher information matrix $I({\theta })$, whose $\{h,j\}$ element is given by

8 $$\begin{aligned} J_{hj}=-E_{{\theta }}\left[ \frac{{\partial }^{2}}{{\partial }{\theta }_{h}{\partial }{\theta }_{j}}\log L({\theta };{\mathbf y} ^{n})\mid {\theta }\right] , \end{aligned}$$

where expectation $E_{{\theta }}$ with respect to the random variable *Y*.

In the life test with Type-II APHCS, notice that the number of removed units at the *i*th $(i=1,2,\dots ,m)$ failure $R_{i}$ is a random variable, then ${\mathbf R} ^{m}$ is a random vector. We suppose that $R_{1},R_{2},\dots ,R_{m}$ are i.i.d., data, and

9 $$\begin{aligned}&R_{i}\thicksim N\left( (n-m)(j+1)^{-1},1\right) ,\quad { for}\,\,y_{j}<\eta <y_{j+1}<y_{m},\end{aligned}$$

10 $$\begin{aligned}&R_{i}\thicksim N\left( (n-m)m^{-1},1\right) ,\quad { for}\,\, y_{m}<\eta , \end{aligned}$$

where $R_{i}(i=1,2,\dots ,m)$ may be not an integer, it contradict with practical, we must become it integer using proper methods in the sense of approximate in the simulation studies.

For convenience, $k=1, 2$, we introduce the following notations:

$$\begin{aligned} {\mathcal {M}}\mathop {=}\limits ^{\mathrm {def}}\left\{ \begin{array}{lll} (n-m)(j+1)^{-1}, &{}\quad Y_{j}<\eta <Y_{j+1}\le Y_{m}, \\ (n-m)m^{-1},&{}\quad Y_{m}<\eta ;\\ \end{array} \right. \end{aligned}$$

$$\begin{aligned}&{\mathscr {L}}({\theta })\mathop {=}\limits ^{\mathrm {def}}\log L({\theta };{\mathbf y} ^{m}),\,\,\,{\partial }_{hj}^{2}{\mathscr {L}}({\theta })\mathop {=}\limits ^{\mathrm {def}} \frac{{\partial }^{2}}{{\partial }{\theta }_{h}{\partial }{\theta }_{j}}\log L({\theta };{\mathbf y} ^{m});\\&{\mathscr {L}}^{D_{k}}_{i}({\theta })\mathop {=}\limits ^{\mathrm {def}}\log f_{k}(y_{i}\mid {\theta }),\,\,\,{\partial }_{hj}^{2}{\mathscr {L}}^{D_{k}}_{i}({\theta }) \mathop {=}\limits ^{\mathrm {def}}\frac{{\partial }^{2}}{{\partial }{\theta }_{h}{\partial }{\theta }_{j}}\log f_{k}(y_{i}\mid {\theta });\\&{\mathscr {L}}^{C_{k}}_{i}({\theta })\mathop {=}\limits ^{\mathrm {def}}\log S_{k}(y_{i}\mid {\theta }),\,\,\,{\partial }_{hj}^{2}{\mathscr {L}}^{C_{k}}_{i}({\theta }) \mathop {=}\limits ^{\mathrm {def}}\frac{{\partial }^{2}}{{\partial }{\theta }_{h}{\partial }{\theta }_{j}}\log S_{k}(y_{i}\mid {\theta });\\&{\mathscr {E}}^{D_{k}}_{\sum }({\theta })\mathop {=}\limits ^{\mathrm {def}}\sum _{i=1}^{m}E_{{\theta }}\left[ {\partial }_{hj}^{2} {\mathscr {L}}^{D_{k}}_{i}({\theta })\mid {\delta }_{1i}=2-k,{\theta }\right] ,\,\,\, {\mathscr {E}}^{D_{k}}({\theta })\mathop {=}\limits ^{\mathrm {def}}E_{{\theta }}\left[ {\partial }_{hj}^{2} {\mathscr {L}}^{D_{k}}_{i}({\theta })\mid {\delta }_{1i}=2-k,{\theta }\right] ;\\&{\mathscr {E}}^{C_{1}}_{\sum }({\theta })\mathop {=}\limits ^{\mathrm {def}}\sum _{i=1}^{m}E_{{\theta }}\left[ R_{i}{\partial }_{hj}^{2} {\mathscr {L}}^{C_{1}}_{i}({\theta })\mid {\delta }_{1i}=1,{\theta }\right] ,\,\,\, {\mathscr {E}}^{C_{1}}({\theta })\mathop {=}\limits ^{\mathrm {def}}E_{{\theta }}\left[ R_{i}{\partial }_{hj}^{2} {\mathscr {L}}^{C_{1}}_{i}({\theta })\mid {\delta }_{1i}=1,{\theta }\right] ; \\&{\mathscr {E}}^{C_{2}}_{\sum }({\theta })\mathop {=}\limits ^{\mathrm {def}}\sum _{i=1}^{m}E_{{\theta }}\left[ R_{i}{\partial }_{hj}^{2} {\mathscr {L}}^{C_{2}}_{i}({\theta })\mid {\delta }_{2i}^{c}=1,{\theta }\right] ,\,\,\, {\mathscr {E}}^{C_{2}}({\theta })\mathop {=}\limits ^{\mathrm {def}}E_{{\theta }}\left[ R_{i}{\partial }_{hj}^{2} {\mathscr {L}}^{C_{2}}_{i}({\theta })\mid {\delta }_{2i}^{c}=1,{\theta }\right] . \end{aligned}$$

### **Theorem 1**

*Let*${\varvec{Y}}^{n}$*be a sample of independent life data, with likelihood function*$L({\theta };{\varvec{y}}^{m})$*given in* (4), *then we have the following results:*

(*a*): $$\begin{aligned} J_{hj}=-E_{{\theta }}\left[ {\partial }_{hj}^{2} {\mathscr {L}}({\theta })\mid {\theta }\right] =-\left( H_{D}({\theta }_{h},{\theta }_{j})+H_{C}({\theta }_{h},{\theta }_{j})\right) , \end{aligned}$$
  *where*
  $$\begin{aligned} H_{D}({\theta }_{h},{\theta }_{j})& = {} {\mathscr {E}}^{D_{1}}_{\sum }({\theta })P\left\{ {\delta }_{1i}=1\mid {\theta }\right\} + {\mathscr {E}}^{D_{2}}_{\sum }({\theta })P\left\{ {\delta }_{1i}=0\mid {\theta }\right\} ,\\ H_{C}({\theta }_{h},{\theta }_{j})& = {} {\mathscr {E}}^{C_{1}}_{\sum }({\theta })P\left\{ {\delta }_{1i}=1\mid {\theta }\right\} +{\mathscr {E}}^{C_{2}}_{\sum }({\theta })P\left\{ {\delta }_{2i}^{c}=1\mid {\theta }\right\} . \end{aligned}$$
(*b*): *If the data are i.i.d., then*
  $$\begin{aligned}&H_{D}({\theta }_{h},{\theta }_{j})=\sum _{k=1,2}\sum _{i=1}^{m}{\mathscr {E}}^{D_{k}}({\theta })P\left\{ {\delta }_{1i}=2-k\mid {\theta }\right\} ,\\&H_{C}({\theta }_{h},{\theta }_{j})={\mathscr {E}}^{C_{1}}({\theta })\sum _{i=1}^{m}P\left\{ {\delta }_{1i}=1\mid {\theta }\right\} + {\mathscr {E}}^{C_{2}}({\theta })\sum _{i=1}^{m}P\left\{ {\delta }_{2i}^{c}=1\mid {\theta }\right\} . \end{aligned}$$

### *Proof*

See “Appendix 1”. $$\square$$

### **Theorem 2**

*Let*${\varvec{Y}}^{n}$*be a sample of independent life data, with likelihood function*$L({\theta };{\varvec{y}}^{n})$*given in* (4), *then*

(*a*): $$J_{hj}=E\left[ n_{u}\mid {\theta }\right] \left( {\mathscr {E}}^{D_{2}}_{\sum }({\theta })+{\mathscr {E}}^{C_{2}}_{\sum }({\theta })- {\mathscr {E}}^{D_{1}}_{\sum }({\theta })-{\mathscr {E}}^{C_{1}}_{\sum }({\theta })\right) -m{\mathscr {E}}^{D_{2}}_{\sum }({\theta })-m{\mathscr {E}}^{C_{2}}_{\sum }({\theta }),$$*if*$$Y_{m}<\eta$$.
(*b*): $$J_{hj}=E\left[ n_{u}\mid {\theta }\right] \left( {\mathscr {E}}^{D_{2}}_{\sum }({\theta })+{\mathscr {E}}^{C_{2}}_{\sum }({\theta })- {\mathscr {E}}^{D_{1}}_{\sum }({\theta })-{\mathscr {E}}^{C_{1}}_{\sum }({\theta })\right) -m{\mathscr {E}}^{D_{2}}_{\sum }({\theta })-(j+1){\mathscr {E}}^{C_{2}}_{\sum }({\theta }),$$*if*$$Y_{j}<\eta <Y_{j+1}<Y_{m}$$.

### *Proof*

See “Appendix 2”. $$\square$$

### **Theorem 3**

*Let*${\varvec{Y}}^{n}$*be a sample of i.i.d., life data, with likelihood function*$L({\theta };{\varvec{y}}^{m})$*given in* (4), *and*$Y_{j}<\eta <Y_{j+1}<Y_{m}$, *then*

$$\begin{aligned} (a)\,\,\, J_{hj}& = {} -{\mathscr {E}}^{D_{1}}({\theta })E\left[ n_{u}\mid {\theta }\right] - {\mathscr {E}}^{D_{2}}({\theta })E\left[ n_{a}\mid {\theta }\right] - {\mathscr {E}}^{C_{1}}({\theta })E\left[ c_{u}\mid {\theta }\right] \\&\quad - E_{{\theta }}\left[ R_{m}{\partial }_{hj}^{2}{\mathscr {L}}_{m}({\theta })\mid {\delta }_{2m}^{c}=1,{\theta }\right] P\left\{ {\delta }_{2m}^{c}=1\mid {\theta }\right\},\,\, if\,\, \eta \le \tau <Y_{m}.\\ (b)\,\,\, J_{hj}& = {} -{\mathscr {E}}^{D_{1}}({\theta })E\left[ n_{u}\mid {\theta }\right] - {\mathscr {E}}^{C_{1}}({\theta })E\left[ c_{u}\mid {\theta }\right],\,\,if\,\, Y_{m}<\tau . \end{aligned}$$

### *Proof*

If the data are i.i.d., from the Theorem 1, the expectation does not depend on index *i*, then

$$\begin{aligned} \sum _{i=1}^{m}E_{{\theta }}\left[ {\partial }^{2}_{hj}{\mathscr {L}}_{i}^{D_{1}}({\theta })\mid {\delta }_{1i} =1,{\theta }\right] P\left\{ {\delta }_{1i}=1\mid {\theta }\right\} ={\mathscr {E}}^{D_{1}}({\theta })\sum _{i=1}^{m}P\left\{ {\delta }_{1i}=1\mid {\theta }\right\} , \end{aligned}$$

that is

$$\begin{aligned} {\mathscr {E}}^{D_{1}}_{\sum }({\theta })={\mathscr {E}}^{D_{1}}({\theta }). \end{aligned}$$

Similarly, we have

$$\begin{aligned} {\mathscr {E}}^{D_{2}}_{\sum }({\theta })={\mathscr {E}}^{D_{2}}({\theta }),\quad {\mathscr {E}}^{C_{k}}_{\sum }({\theta })={\mathscr {E}}^{C_{k}}({\theta })\,\,(k=1,2). \end{aligned}$$

If $\eta \le \tau <Y_{m}$, then the censored data only valid at time $Y_{m}$. If $Y_{m}<\tau$, then the accelerated stress is invalid. The results can be shown via a standard computation. $\square$

Based on the Theorem 3, we get the following results.

### **Corollary 1**

*Let*$${\varvec{Y}}^{n}$$*be a sample of i.i.d., life data, with likelihood function*$$L({\theta };{\varvec{y}}^{m})$$*given in* (4), $$C_{k}=\emptyset$$$$(k=1,2)$$, *then*

$$\begin{aligned} (a)\,\,\, J_{hj}& = {} -{\mathscr {E}}^{D_{1}}({\theta })E\left[ n_{u}\mid {\theta }\right] - {\mathscr {E}}^{D_{2}}({\theta })E\left[ n_{a}\mid {\theta }\right] , \quad if\,\, \eta \le \tau <Y_{m}.\\ (b)\,\,\, J_{hj}& = {} -{\mathscr {E}}^{D_{1}}({\theta })E\left[ n_{u}\mid {\theta }\right] ,\quad if\; Y_{m}<\tau . \end{aligned}$$

### **Corollary 2**

*Let*$${\varvec{Y}}^{n}$$*be a sample of i.i.d., life data, with likelihood function*$$L({\theta };{\varvec{y}}^{n})$$*given in* (4), $$\dim ({\theta })=p$$*and*$$C_{k}=\emptyset$$$$(k=1,2)$$, *then the Jeffreys prior is*

$$\begin{aligned} \pi ^{J}({\theta })=\widetilde{\pi }^{J}({\theta })\left( E[n_{u}\mid {\theta }]\right) ^{p/2}, \end{aligned}$$

*where*$\widetilde{\pi }^{J}({\theta })$*is the Jeffreys prior for the uncensored case under normal conditions.*

### Jeffreys priors for the Weibull distribution

In this section, we investigate the Jeffreys priors for the Weibull distribution. Suppose *n* independent units are placed on a life test, and ${\mathbf Y} ^{n}$ denotes a Type-II APHCS sample from the Weibull distribution with shape and scale parameters as ${\beta }$ and ${\theta }$ respectively, the probability density function (pdf) of *Y* is given by

11 $$\begin{aligned} f_{Y}(y;{\theta },{\beta })={\beta }y^{{\beta }-1}{\theta }^{-{\beta }}\exp \{-(y/{\theta })^{{\beta }}\},\quad y>0,\,\, {\theta }>0,\,\, {\beta }>0. \end{aligned}$$

In the uncensored data and without accelerated stress setting, Sun (1997) proved that the Jeffreys prior for the Weibull density function is

12 $$\begin{aligned} \pi _{J}({\theta },{\beta })\propto 1/{\theta }. \end{aligned}$$

In order to obtain the exact results, in this section, we assume that the censoring scheme is *b*. Based on the Balakrishnan and Kundu (2013), the likelihood function under SSPALT with Type II APHCS can be provided as follows

13 $$\begin{aligned} L({\theta },{\beta }; {\mathbf y} ^{m}) =\prod _{i=1}^{n_{u}}f_{1}(y_{i})S_{1}^{R_{i}}(y_{i}) \prod _{i=n_{u}+1}^{j}f_{2}(y_{i})S_{2}^{R_{i}}(y_{i}) \prod _{i=j+1}^{m}f_{2}(y_{i})S_{2}^{R_{m}}(y_{m}). \end{aligned}$$

Let $Y_{a}=\tau +k(Y-\tau )$, then the density function of $Z_{a}=\left( Y_{a}/{\theta }\right) ^{{\beta }}$ can be obtained via a standard computation, that is

$$\begin{aligned} f(z_{a})\propto {\theta }^{-{\beta }+1}z_{a}^{\frac{1}{{\beta }}-1}\left( k^{-1}({\theta }z_{a}^{{\beta }^{-1}}-\tau ) +\tau \right) ^{{\beta }-1}\exp \left\{ -{\theta }^{-{\beta }}\left( k^{-1}({\theta }z_{a}^{{\beta }^{-1}}-\tau )+\tau \right) ^{{\beta }}\right\} . \end{aligned}$$

Observe that the density function of $z_{a}$ is complicated, for simplicity, we also introduce the notations

14 $$\begin{aligned} {\mathscr {E}}^{v,w}={\beta }^{w}E_{({\theta },{\beta })}\left[ \left( \frac{y_{a}}{{\theta }}\right) ^{{\beta }v} \left[ \ln \left( \frac{y_{a}}{{\theta }}\right) \right] ^{w}\right] =E_{({\theta },{\beta })}\left[ z_{a}^{ v} (\ln z_{a})^{w}\right] ,\quad v,w=0,1,2, \end{aligned}$$

provided that the integral exists, where $E_{({\theta },{\beta })}$ means the expectation being with respect to the random variable $Z_{a}$.

If the acceleration factor $k=1$, that is $Y=Y_{a}$, $Z=(Y/{\theta })^{{\beta }}$ is an exponential random variable with mean 1. For $u\ge 1$, let

15 $$\begin{aligned} \gamma _{u}=\int _{0}^{\infty }[\log (z)]^{u}e^{-z}dz \end{aligned}$$

be the *u*th moment of $\log (Z)$, we have the following relationships between ${\gamma }$ and $\mathscr {E}$:

- $${\mathscr {E}}^{1,0}=1,\quad {\mathscr {E}}^{0,1}={\gamma }_{1}$$,
- $${\mathscr {E}}^{1,1}=1+{\gamma }_{1},\quad {\mathscr {E}}^{1,2}={\gamma }_{2}+2{\gamma }_{1},$$

where $-{\gamma }_{1}$ is Euler’s constant, ${\gamma }_{2}-{\gamma }_{1}^{2}$ is the variance of $\log (Z)$. After a standard computation, some results can be obtained as follows.

#### **Lemma 1**

*From the density function* (11) *and the likelihood function* (13), *we have*

$$\begin{aligned}&J_{20}={\theta }^{-2}\left( m{\beta }-{\beta }({\beta }+1)(n_{u}({\mathcal {M}}+1)+ ({\mathcal {M}}c_{a}+n_{a}){\mathscr {E}}^{1,0})\right) ;\\&J_{02}={\beta }^{-2}\left( -m-n_{u}({\mathcal {M}}+1)({\gamma }_{2}+2{\gamma }_{1})- ({\mathcal {M}}c_{a}+n_{a}){\mathscr {E}}^{1,2}\right) ;\\&J_{11}={\theta }^{-1}\left( -m+n_{u}({\mathcal {M}}+1)(2+{\gamma }_{1})+ ({\mathcal {M}}c_{a}+n_{a})({\mathscr {E}}^{1,1}+{\mathscr {E}}^{1,0})\right) . \end{aligned}$$

#### *Proof*

See “Appendix 3”. $$\square$$

Therefore, the expected Fisher information matrix of $({\theta },{\beta })$ is

$$\begin{aligned} \sum =-\left( \begin{array}{cc} J_{20} &{} J_{11} \\ J_{11} &{} J_{02} \\ \end{array} \right) , \end{aligned}$$

and the determinant of $\Sigma$ is

$$\begin{aligned} \det \left( \Sigma \right) ={\theta }^{-2}\psi _{1}({\beta }), \end{aligned}$$

where

$$\begin{aligned}\psi _{1}({\beta })& = {} \left( -m{\beta }^{-1}+{\beta }^{-1}({\beta }+1)(n_{u}({\mathcal {M}}+1)+ ({\mathcal {M}}c_{a}+n_{a}){\mathscr {E}}^{1,0})\right) \\&\quad \times \left( m+n_{u}({\mathcal {M}}+1)({\gamma }_{2}+2{\gamma }_{1})+ ({\mathcal {M}}c_{a}+n_{a}){\mathscr {E}}^{1,2}\right) \\&\quad -\left( m-n_{u}({\mathcal {M}}+1)(2+{\gamma }_{1})- ({\mathcal {M}}c_{a}+n_{a})({\mathscr {E}}^{1,1}+{\mathscr {E}}^{1,0})\right) ^{2}. \end{aligned}$$

The following result can be immediately obtained.

#### **Theorem 4**

*Let*${\varvec{Y}}^{n}$*be the failure times observed from Weibull*$({\theta },{\beta })$, *then the Jeffreys prior based on the SSPALT with Type-II APHCS is given by*

$$\begin{aligned} \pi _{1J}({\theta },{\beta })\propto {\theta }^{-1}\sqrt{|\psi _{1}({\beta })|}, \end{aligned}$$

*where*$\psi _{1}({\beta })>0$*is a constraint which may not be satisfied in practice.*

A obvious fact is that the Jeffreys prior depends on the accelerated stress levels, the number of observed units and the number of censored items. But how about without accelerated stress? In this case, $n_{a}=c_{a}=0,n_{u}=m$, then

$$\begin{aligned} J_{20}& = {} {\theta }^{-2}\left( m{\beta }-{\beta }({\beta }+1)m({\mathcal {M}}+1)\right) ;\\ J_{02}& = {} {\beta }^{-2}\left( -m-m({\mathcal {M}}+1)({\gamma }_{2}+2{\gamma }_{1}\right) ;\\ J_{11}& = {} {\theta }^{-1}\left( -m+m({\mathcal {M}}+1)(2+{\gamma }_{1})\right) . \end{aligned}$$

#### **Theorem 5**

*Let*${\varvec{Y}}^{n}$*be the failure times observed from Weibull*$({\theta },{\beta })$, *then the Jeffreys prior with the Type-II APHCS is given by*

$$\begin{aligned} \pi _{2J}({\theta },{\beta })\propto m{\theta }^{-1}\sqrt{|\psi _{2}({\beta })|}, \end{aligned}$$

*where*$$\psi _{2}({\beta })=({\mathcal {M}}+1+{\mathcal {M}}{\beta }^{-1})(1+({\mathcal {M}}+1)({\gamma }_{2}+2{\gamma }_{1})) -(1-({\mathcal {M}}+1)({\gamma }_{1}+2))^{2}>0$$.

If the experimenter dose not remove the sample at each failure time except the *m*th failure, that is Type-II CS, then $n_{u}=m$, $n_{u}{\mathcal {M}}=R_{m}$, we have

$$\begin{aligned}&J_{20}={\theta }^{-2}\left( -m{\beta }^{2}-R_{m}{\beta }({\beta }+1)\right) ;\\&J_{02}={\beta }^{-2}\left( -m-(m+R_{m})({\gamma }_{2}+2{\gamma }_{1})\right) ;\\&J_{11}={\theta }^{-1}\left( m(1+{\gamma }_{1})+ R_{m}(2+{\gamma }_{1})\right) . \end{aligned}$$

The following results are obtained directly.

#### **Theorem 6**

*Let*${\varvec{Y}}^{n}$*be the failure times observed from Weibull*$({\theta },{\beta })$, *then the Jeffreys prior with the Type-II CS is given by*

$$\begin{aligned} \pi _{3J}({\theta },{\beta })\propto {\theta }^{-1}\sqrt{|\psi _{3}({\beta })|}, \end{aligned}$$

*where*$$\psi _{3}({\beta })=(m+R_{m}+R_{m}{\beta }^{-1})(m+(m+R_{m})({\gamma }_{2}+2{\gamma }_{1})) -(m(1+{\gamma }_{1})+R_{m}({\gamma }_{1}+2))^{2}>0$$.

Considering the censored data does not presented in the life test, then $m=n, R_{m}=0$.

#### **Corollary 3**

*Let*${\varvec{Y}}^{n}$*be the failure times observed from Weibull*$({\theta },{\beta })$, *then the Jeffreys prior with complete sample is given by*

$$\begin{aligned} \pi _{4J}({\theta },{\beta })\propto n{\theta }^{-1}\sqrt{|\psi _{4}|}\propto {\theta }^{-1}, \end{aligned}$$

*where*$\psi _{4}={\gamma }_{2}-{\gamma }_{1}^{2}$*is the variance of*$\log (Z)$, $Z\sim \exp (1)$.

### Posterior analysis

Suppose ${\mathbf Y} ^{n}$ is a sample from Weibull $({\theta },{\beta })$ under the SSPALT with Type-II APHCS. Let

- $$\sum _{i\in [1:n_{u}:j:m]}(y_{i},y_{ai},R_{i},{\beta }) \mathop {=}\limits ^{\mathrm {def}}\sum _{i=1}^{n_{u}}(1+R_{i})y_{i}^{{\beta }}+\sum _{i=n_{u}+1}^{j}(1+R_{i})y_{ai}^{{\beta }} +\sum _{i=j+1}^{m}y_{ai}^{{\beta }}+R_{m}y_{am}^{{\beta }};$$
- $$\prod _{i\in [1:n_{u}:m]}(y_{i},y_{ai},{\beta }) \mathop {=}\limits ^{\mathrm {def}}\prod _{i=1}^{n_{u}}y_{i}^{{\beta }-1}\prod _{i=n_{u}+1}^{m}y_{ai}^{{\beta }-1};$$
- $$m_{1}\mathop {=}\limits ^{\mathrm {def}}n_{u}({\mathcal {M}}+1)+ ({\mathcal {M}}c_{a}+n_{a}){\mathscr {E}}^{1,0}$$;
- $$m_{2}\mathop {=}\limits ^{\mathrm {def}}m+n_{u}({\mathcal {M}}+1)({\gamma }_{2}+2{\gamma }_{1})+ ({\mathcal {M}}c_{a}+n_{a}){\mathscr {E}}^{1,2}$$.

Notice that $n_{u}+n_{a}=m$, it is clear that $m_{1}>m$, $\sqrt{\psi _{1}({\beta })}\le m_{1}m_{2}+m_{2}(m_{1}-m){\beta }^{-1}$.

Let

$$\begin{aligned} Z_{i}=\left\{ \begin{array}{lll} Y_{i}, &{}\quad if \,\,i=1,\dots ,n_{u}, \\ Y_{ai},&{}\quad if \,\,i=n_{u}+1,\dots ,m.\\ \end{array} \right. \end{aligned}$$

If there is $Z_{k}$ such that $Z_{k}\le \max \{Z_{1},\dots ,Z_{m}\}$, $m\ge 2$, and the observations are distinct. Then from proposition 1 in Sun (1997), we have

$$\begin{aligned}&\int _{0}^{\infty }\int _{0}^{\infty }L({\mathbf{data} };{\theta },{\beta })\pi _{1J}({\theta },{\beta })d{\theta }d{\beta }\\&\quad \le k^{c_{a}+n_{a}}\Gamma (m)\int _{0}^{\infty }{\beta }^{m-1}\left( m_{2}m_{1}+\frac{m_{2}(m_{1}-m)}{{\beta }}\right) \left( \frac{Z_{k}}{\max \{Z_{1},\dots ,Z_{m}\}}\right) ^{{\beta }}d{\beta }\\&\quad \le \infty . \end{aligned}$$

Similarly, we can check the following result.

#### **Theorem 7**

*If*$m\ge 2$*and*$Y_{1},\dots ,Y_{n_{u}},Y_{n_{u}+1},\dots ,Y_{m}$*are distinct, then the posterior distribution of*$({\theta },{\beta })$*based on the Jeffreys priors*$\pi _{1J},\pi _{2J},\pi _{3J},\pi _{4J}$*are proper, respectively.*

Under this theorem conditions, the following results can be arrived via a standard computation.

#### **Theorem 8**

*Given the Jeffreys prior*$\pi _{1J}$*based on the SSPALT with Type-II APHCS, then*

(*a*): *the marginal posterior density of*${\theta }$*is given by*
  $$\begin{aligned} h_{11}({\theta }|{{\varvec{data}}})=\frac{1}{c_{1}}\int _{0}^{\infty }\frac{{\beta }^{m}\sqrt{\psi _{1}({\beta })}}{{\theta }^{m{\beta }+1}} \prod _{i\in [1:n_{u}:m]}(y_{i},y_{ai},{\beta })\exp \left\{ -\frac{\sum _{i\in [1:n_{u}:j:m]} (y_{i},y_{ai},R_{i},{\beta })}{{\theta }^{{\beta }}}\right\} d{\beta }; \end{aligned}$$
(*b*): *the marginal posterior cumulative distribution function of*${\theta }$*is given by*
  $$\begin{aligned} H_{11}({\theta }|{{\varvec{data}}})& = {} \frac{1}{c_{1}}\int _{0}^{\infty }s^{m-1}\sqrt{\psi _{1}(s)} \prod _{i\in [1:n_{u}:m]}(y_{i},y_{ai},s)\left( \sum _{i\in [1:n_{u}:j:m]} (y_{i},y_{ai},R_{i},s)\right) ^{-m}\\&\quad \times I_{\Gamma }\left( m,\frac{\sum _{i\in [1:n_{u}:j:m]} (y_{i},y_{ai},R_{i},s)}{{\theta }^{s}}\right) ds; \end{aligned}$$
(*c*): *the marginal posterior density of*${\beta }$*is given by*
  $$\begin{aligned} h_{12}({\beta }|{{\varvec{data}}})=\frac{1}{c_{1}}{\beta }^{m-1}\sqrt{\psi _{1}({\beta })}\Gamma (m) \prod _{i\in [1:n_{u}:m]}(y_{i},y_{ai},{\beta })\left( \sum _{i\in [1:n_{u}:j:m]} (y_{i},y_{ai},R_{i},{\beta })\right) ^{-m}; \end{aligned}$$
(*d*): *the marginal posterior cumulative distribution function of*${\beta }$*is given by*
  $$\begin{aligned} H_{12}({\beta }|{{\varvec{data}}})=\frac{1}{c_{1}}\Gamma (m)\int _{0}^{{\beta }}s^{m-1}\sqrt{\psi _{1}(s)} \prod _{i\in [1:n_{u}:m]}(y_{i},y_{ai},s)\left( \sum _{i\in [1:n_{u}:j:m]} (y_{i},y_{ai},R_{i},s)\right) ^{-m}ds. \end{aligned}$$
  *where*
  $$\begin{aligned} c_{1}\mathop {=}\limits ^{\mathrm {def}}\Gamma (m)\int _{0}^{\infty }s^{m-1}\sqrt{\psi _{1}(s)} \prod _{i\in [1:n_{u}:m]}(y_{i},y_{ai},s)\left( \sum _{i\in [1:n_{u}:j:m]} (y_{i},y_{ai},R_{i},s)\right) ^{-m}ds \end{aligned}$$
  *is the normalizing constant,*$I_{\Gamma }(m,y)\mathop {=}\limits ^{\mathrm {def}}\int _{y}^{\infty }s^{m-1}\exp \{-s\}ds$*is the complementary incomplete Gamma function and*$\Gamma (\cdot )$*is the Gamma function.*

## Permissible Jeffreys priors

Permissible priors, as pointed by Berger et al. (2014), can be viewed as some objective priors to those that satisfy the expected logarithmic convergence condition. We first recall the following definitions, for more details, we refer to Kullback and Leibler (1951), and Berger et al. (2009).

### **Definition 1**

(Kullback and Leibler 1951) The logarithmic divergence of a probability density $\widetilde{p}(y)$ of the random vector $y\in \mathcal {Y}$ from its true probability density *p*(*y*), denoted by

$$\begin{aligned} {\mathcal {K}}\{\widetilde{p}\mid p\}=\int _{\mathcal {Y}}p(y)\log \left\{ \frac{p(y)}{\widetilde{p}(y)}\right\} dy \end{aligned}$$

provided the integral (or the sum) is finite.

${\mathcal {K}}\{\widetilde{p}\mid p\}$ given a method that how to measure the distance between the distribution *p* and $\widetilde{p}$. It is clear that ${\mathcal {K}}\{\widetilde{p}\mid p\}$ does not the normal in the meaning of functional analysis because it may be ${\mathcal {K}}\{\widetilde{p}\mid p\}\ne {\mathcal {K}}\{p\mid \widetilde{p}\}$. Berger et al. (2014) suggested that ${\mathcal {K}}\{\widetilde{p}\mid p\}$ is a divergence, not a distance, a generalized divergence can be found in reference Bernardo (2005) where the divergence is equipped with both primary advantages and normal benefits.

### **Definition 2**

(Berger et al. 2009) Consider a parametric model $\{p(y\mid {\theta }),y\in {\mathcal {Y}},{\theta }\in \Theta \}$, a strictly positive continuous function $\pi ({\theta }),{\theta }\in \Theta$, and an approximating compact sequence $\{\Theta _{i}\}_{i=1}^{\infty }$ of parameter spaces. The corresponding sequence of posteriors $\{\pi _{i}({\theta }\mid y)\}_{i=1}^{\infty }$ is said to be expected logarithmically convergent to the formal posterior $\pi ({\theta }\mid y)$ if

$$\begin{aligned} \lim \limits _{i{\rightarrow }\infty }\int _{\mathcal {X}}{\mathcal {K}}\{\pi (\cdot \mid y)\mid \pi _{i}(\cdot \mid y)\}p_{i}(y)dy=0 \end{aligned}$$

where $$p_{i}(y)=\int _{\Theta _{i}}p(y\mid {\theta })\pi _{i}({\theta })d{\theta }$$.

### **Definition 3**

(Berger et al. 2009) A strictly positive continuous function $\pi ({\theta })$ is a permissible prior for model $\{p(y\mid {\theta }),y\in {\mathcal {Y}},{\theta }\in \Theta \}$ if

1. for all $y\in \mathcal {Y}$, $\pi ({\theta }|y)$ is proper, that is $\int _{\Theta }p(y|{\theta })\pi ({\theta })d{\theta }<\infty$.
2. for some approximating compact sequence, the corresponding posterior sequence is expected logarithmically convergent to $\pi ({\theta }|y)\propto p(y|{\theta })\pi ({\theta })$.

Observe that ${\theta }$ is a scale parameter of $Weibull({\theta },{\beta })$, using the results of Corollary 2 in Berger et al. (2009), we have

$$\begin{aligned} \lim _{|t|{\rightarrow }\infty }|t|^{1+{\varepsilon }}e^{t}f(e^{t})=\lim _{|t|{\rightarrow }\infty }|t|^{1+{\varepsilon }}\exp \{t\} {\beta }\exp \{t({\beta }-1)\}{\theta }^{-{\beta }}\exp \left\{ -\left( e^{t}{\theta }^{-1}\right) ^{{\beta }}\right\} =0, \end{aligned}$$

where ${\varepsilon }>0$ is a constant number, then we have $\pi ({\theta })={\theta }^{-1}$ is a permissible prior function for the Weibull $({\theta },{\beta })$ probability density function.

As suggested Berger et al. (2009), a prior might be permissible for a larger sample size, even if it is not permissible for a minimal sample size. But if a prior permissible for the minimal sample size, can we obtain it is permissible for the larger sample size? The answer is positive. In fact, there exists a relationship between single observation and multi observations concerning with the permissibility of a prior. This relationship first illustrated by Berger et al. (2009), and based on which, we have the following theorem.

### **Theorem 9**

*Let*$\{p({\varvec{y}}^{n}\mid {\theta })=p({\varvec{y}}^{k}\mid {\varvec{y}}^{k+1,n},{\theta })p({\varvec{y}}^{k+1,n}\mid {\theta }),k=1,2,\dots ,n,y_{i}\in {\mathcal {Y}}_{i}\subset {\mathcal {Y}},{\theta }\in \Theta \}$*be a likelihood function family. Consider a continuous improper prior*$\pi ({\theta })$*satisfying*

$$\begin{aligned} m({\varvec{y}}^{k}\mid {\varvec{y}}^{k+1,n})=\int _{\Theta }p({\varvec{y}}^{k}\mid {\varvec{y}}^{k+1,n},{\theta })\pi ({\theta })d{\theta }<\infty ,\quad k=1,2,\dots ,n. \end{aligned}$$

*For any compact set*$\Theta _{0}\subset \Theta$, $\pi _{0}({\theta })=\frac{\pi ({\theta }){\varvec{I}}_{\Theta _{0}}}{\int _{\Theta _{0}}\pi ({\theta })d{\theta }}$, *then*

$$\begin{aligned}&\idotsint \limits _{\prod _{i=1}^{n}{\mathcal {Y}}_{i}}{\mathcal {K}}\left\{ \pi ({\theta }\mid {\varvec{y}}^{n})\mid \pi _{0}({\theta }\mid {\varvec{y}}^{n})\right\} m_{0}({\varvec{y}}^{n})d{\varvec{y}}^{n} \\&\quad \le \idotsint \limits _{\prod _{i=1}^{n-1}{\mathcal {Y}}_{i}}{\mathcal {K}}\left\{ \pi ({\theta },y_{n}\mid {\varvec{y}}^{n-1})\mid \pi _{0}({\theta },y_{n}\mid {\varvec{y}}^{n-1})\right\} m_{0}({\varvec{y}}^{n-1}\mid y_{n})d{\varvec{y}}^{n-1}\\&\qquad \vdots \\&\quad \le \idotsint \limits _{\prod _{i=1}^{k}{\mathcal {X}}_{i}}{\mathcal {K}}\left\{ \pi ({\theta },{\varvec{y}}^{k+1,n}\mid {\varvec{y}}^{k})\mid \pi _{0}({\theta },{\varvec{y}}^{k+1,n}\mid {\varvec{y}}^{k})\right\} m_{0}({\varvec{y}}^{k}\mid {\varvec{y}}^{k+1,n})d{\varvec{y}}^{k} \\&\qquad \vdots \\&\quad \le \int \limits _{{\mathcal {Y}}_{1}}{\mathcal {K}}\left\{ \pi ({\theta },{\varvec{y}}^{2,n}\mid y_{1})\mid \pi _{0}({\theta },{\varvec{y}}^{2,n}\mid y_{1})\right\} m_{0}(y_{1}\mid {\varvec{y}}^{2,n})dy_{1}, \end{aligned}$$

*where*

$$\begin{aligned}&\pi _{0}({\theta }, {\varvec{y}}^{k,n}\mid {\varvec{y}}^{k-1})=\frac{p( {\varvec{y}}^{k-1}\mid {\varvec{y}}^{k,n},{\theta })\pi ({\theta })}{m_{0}( {\varvec{y}}^{k-1}\mid {\varvec{y}}^{k,n})},\\&m_{0}( {\varvec{y}}^{k-1}\mid y_{k},\dots , y_{n})=\int _{\Theta _{0}}p( {\varvec{y}}^{k-1}\mid {\varvec{y}}^{k,n},{\theta })\pi ({\theta })d{\theta },\quad k=1,2,\dots ,n. \end{aligned}$$

### *Proof*

See “Appendix 4”. $$\square$$

Theorem 9 guarantees that the Jeffreys prior $\pi ({\theta })={\theta }^{-1}$ is a permissible prior function for the multi observations. This theorem also reveals that the expected logarithmic discrepancy is monotonically non-increasing in sample size, but how much they difference, the following corollary gives exact answer.

### **Corollary 4**

*Let*${\mathcal {Y}}$*be a sample space,*$\{p({\varvec{y}}^{n}\mid {\theta })=p({\varvec{y}}^{k}\mid {\varvec{y}}^{k+1,n},{\theta })p({\varvec{y}}^{k+1,n}\mid {\theta }),k=1,2,\dots ,n,y_{i}\in {\mathcal {Y}}_{i}\subset {\mathcal {Y}},{\theta }\in \Theta \}$*be a likelihood function family. Consider a continuous improper prior*$\pi ({\theta })$*satisfying*

$$\begin{aligned} m({\varvec{y}}^{k}\mid {\varvec{y}}^{k+1,n})=\int _{\Theta }p({\varvec{y}}^{k}\mid {\varvec{y}}^{k+1,n},{\theta })\pi ({\theta })d{\theta }<\infty . \end{aligned}$$

*For any compact set*$\Theta _{0}\subset \Theta$, $\pi _{0}({\theta })=\frac{\pi ({\theta }){\varvec{I}}_{\Theta _{0}}}{\int _{\Theta _{0}}\pi ({\theta })d{\theta }}$, *then*

$$\begin{aligned}&\idotsint \limits _{\prod _{i=1}^{k}{\mathcal {Y}}_{i}}{\mathcal {K}}\left\{ \pi ({\theta },{\varvec{y}}^{k+1,n}\mid {\varvec{y}}^{k})\mid \pi _{0}({\theta },{\varvec{y}}^{k+1,n}\mid {\varvec{y}}^{k})\right\} m_{0}({\varvec{y}}^{k}\mid {\varvec{y}}^{k+1,n})d{\varvec{y}}^{k} \\&\qquad - \idotsint \limits _{\prod _{i=1}^{k-1}{\mathcal {Y}}_{i}}{\mathcal {K}}\left\{ \pi ({\theta },{\varvec{y}}^{k,n}\mid {\varvec{y}}^{k-1})\mid \pi _{0}({\theta },{\varvec{y}}^{k,n}\mid {\varvec{y}}^{k-1})\right\} m_{0}({\varvec{y}}^{k-1}\mid {\varvec{y}}^{k,n})d{\varvec{y}}^{k-1}\\&\quad =-\int _{{\mathcal {Y}}_{k}}\log \left\{ \frac{m(y_{k}\mid {\varvec{y}}^{k+1,n})}{m_{0}(y_{k}\mid {\varvec{y}}^{k+1,n})}\right\} m_{0}(y_{k}\mid {\varvec{y}}^{k+1,n})dy_{k}, \end{aligned}$$

*where*$\pi _{0}({\theta }, {\varvec{y}}^{k,n}\mid {\varvec{y}}^{k-1}),m_{0}( {\varvec{y}}^{k-1}\mid {\varvec{y}}^{k,n})$*are same as in Theorem*9.

## Simulation studies and frequency analysis

Coverage probabilities are used to value a prior good or bad. The idea, as suggested by Ye (1993), is that if prior $\pi _{1}$ has generally smaller difference between the posterior probabilities of Bayesian credible sets and the frequentist probabilities of the corresponding confidence sets than does $\pi _{2}$, then prior $\pi _{1}$ is favorable.

Let $Y\sim Weibull({\theta },{\beta })$, given the Jeffreys prior $\pi _{1J}$ based on the SSPALT with Type-II APHCS data, it is can be seen that the joint posterior density function is very complicated. Samples of ${\theta }$ and ${\beta }$ cannot be generated analytically to well known distributions, so sample directly by standard methods may be difficult. Now we resort to the hybrid algorithm, which introduced by Tierney (1994), by combining Metropolis sampling with the Gibbs sampling scheme using normal proposal distribution. Solimana et al. (2012) referred to the algorithm as hybrid MCMC method.

Notice that the full posterior conditional distributions of ${\theta }$ and ${\beta }$ are given by, respectively

16 $$\begin{aligned} \pi _{1}({\theta }|{\beta },{\mathbf{data }})& = {} \frac{1}{{\theta }^{m{\beta }+1}} \exp \left\{ -\frac{\Sigma _{i\in [1:n_{u}:j:m]} (y_{i},y_{ai},R_{i},{\beta })}{{\theta }^{{\beta }}}\right\} , \end{aligned}$$

17 $$\begin{aligned} \pi _{2}({\beta }|{\theta },{\mathbf{data }})& = {} \frac{{\beta }^{m}\sqrt{\psi _{1}({\beta })}}{{\theta }^{-m{\beta }}} \prod _{i\in [1:n_{u}:m]}(y_{i},y_{ai},{\beta })\exp \left\{ -\frac{\Sigma _{i\in [1:n_{u}:j:m]} (y_{i},y_{ai},R_{i},{\beta })}{{\theta }^{{\beta }}}\right\} , \end{aligned}$$

where $\sum$, $\prod$, $\psi _{1}$ can be found in “Jeffreys priors for the Weibull distribution” section, $y_{i}(i=1,\ldots ,m)$ are Type-II progressive censoring samples generated by using the algorithm presented in Balakrishnan and Sandhu (1995) and Balakrishnan and Aggarwala (2000).

**Fig. 1:**
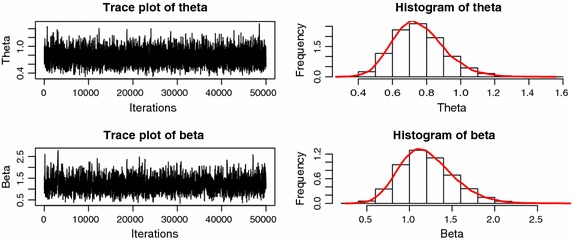
Markov chains’ trace plots and histograms of ${\theta }$ and ${\beta }$ under use normal condition

**Fig. 2:**
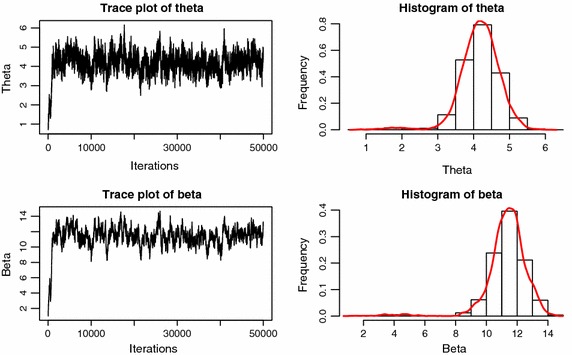
Markov chains’ trace plots and histograms of ${\theta }$ and ${\beta }$ with accelerated factor $k=2$

Given the current value of ${\theta }^{(j-1)}$, to sample ${\theta }$ from (16), a proposal value ${\theta }^{'}$ can be obtained from $N({\theta }^{(j-1)},K_{{\theta }}V_{{\theta }})$, then we renew the ${\theta }^{(j-1)}$ by a probability, where $V_{{\theta }}$ and $K_{{\theta }}$ are variances-covariances matrix and scaling factor that adjust the rate of rejected or accepted samples. Similarly, given the current value of ${\beta }^{(j-1)}$, to sample ${\beta }$ from (17), a proposal value ${\beta }^{'}$ can be obtained from $N({\beta }^{(j-1)},K_{{\beta }}V_{{\beta }})$, then we renew the ${\beta }^{(j-1)}$ by a probability. Robert et al. (1996) suggested that the reject rate in [0.15, 0.5] may be yields a well result. Specifically, this algorithm can be described as follows:

1. Given the current values of ${\theta }^{(j-1)}$ and ${\beta }^{(j-1)}$.
2. Using Metropolis random walk algorithm, generate ${\theta }^{(j)}$ from $\pi _{1}({\theta }^{(j-1)}|{\beta }^{(j-1)},{\mathbf{data} })$ with normal proposal distribution $N({\theta }^{(j-1)},K_{{\theta }}V_{{\theta }})$.
  2*a*. Simulate a candidate value ${\theta }^{'}$ from the proposal density $N({\theta }^{(j-1)},K_{{\theta }}V_{{\theta }})$.
  2*b*. Compute the ratio $r_{1}=\frac{\pi _{1}({\theta }^{'}|{\beta }^{(j-1)},{\mathbf{data} })}{\pi _{1}({\theta }^{(j-1)}|{\beta }^{(j-1)},{\mathbf{data} })}$.
  2*c*. Compute the acceptance probability $p_{1}=\min \{1,r_{1}\}$.
  2*d*. Sample a value ${\theta }^{(j)}$ such that ${\theta }^{(j)}={\theta }^{'}$ with probability $p_{1}$, otherwise ${\theta }^{(j)}={\theta }^{(j-1)}$.
3. Employing Metropolis random walk algorithm, generate ${\beta }^{(j)}$ from $\pi _{2}({\beta }^{(j-1)}|{\theta }^{(j)},{\mathbf{data} })$ with normal proposal distribution $N({\beta }^{(j-1)},K_{{\beta }}V_{{\beta }})$.
  3*a*. Simulate a candidate value ${\beta }^{'}$ from the proposal density $N({\beta }^{(j-1)},K_{{\beta }}V_{{\beta }})$.
  3*b*. Compute the ratio $r_{2}=\frac{\pi _{2}({\beta }^{'}|{\theta }^{(j)},{\mathbf{data} })}{\pi _{2}({\beta }^{(j-1)}|{\theta }^{(j)},{\mathbf{data} })}$.
  3*c*. Compute the acceptance probability $p_{2}=\min \{1,r_{2}\}$.
  3*d*. Sample a value ${\beta }^{(j)}$ such that ${\beta }^{(j)}={\theta }^{'}$ with probability $p_{2}$, otherwise ${\beta }^{(j)}={\beta }^{(j-1)}$.
4. Repeat the steps *N* times.

Let sample size $n=30$, censoring scheme $R=(0,1,1,2,1,1,2,3,1,2,2,2)$, stress change time is the 7th unit failure time, parameter true values $({\theta },{\beta })=(1,1)$. We run this algorithm to generate a Markov chain with 50,000 observations. Discarding the first 500 values as burn-in period. Figures 1 and 2 are the outputs of the Markov chain under use normal condition and accelerated condition, respectively. It is clear that the chains are convergence well. The reject rates are about 0.19, 0.22 in Fig. 1 for ${\theta }$, ${\beta }$, and 0.37, 0.41 in Fig. 2 for ${\theta }$, ${\beta }$, respectively.

**Table 1:** Frequentist coverage probabilities for ${\alpha }=0.95,0.05$ with true parameters ${\theta }=1,{\beta }=1$, and without considering the hybrid censoring time $\eta$

Censoring scheme					$Q_{\pi _{1}}({\theta })$		$Q_{\pi _{1}}({\beta })$	
n	m	$$\left( \begin{array}{cccc} R_{1}&{} R_{2} &{}\cdots \, R_{n_{u}-1}\\ R_{n_{u}} &{}\cdots &{}R_{m} \\ \end{array} \right)$$			${\alpha }=0.95$	${\alpha }=0.05$	${\alpha }=0.95$	${\alpha }=0.05$
10	6	$$\left( \begin{array}{ccc} 0&{} 1 &{} 1 \\ 1 &{} 0 &{} 1 \\ \end{array} \right)$$	k = 1		0.9756	0.0763	0.9233	0.0752
			k = 2	$\tau =4$	0.9137	0.0869	0.9149	0.0865
16	8	$$\left( \begin{array}{cccc} 1&{} 0 &{} 1 &{} 0 \\ 2 &{} 1 &{} 2 &{}1 \\ \end{array} \right)$$	k = 1		0.9314	0.0321	0.9318	0.0686
			k = 2	$\tau =5$	0.9221	0.0764	0.9768	0.0786
24	10	$$\left( \begin{array}{ccccc} 1&{} 0 &{} 1&{} 1&{} 1 \\ 2 &{} 1 &{} 2&{} 2&{} 3 \\ \end{array} \right)$$	k = 1		0.9378	0.0621	0.9629	0.0384
			k = 2	$\tau =6$	0.9705	0.0692	0.9304	0.0302
30	12	$$\left( \begin{array}{cccccc} 0&{} 1 &{} 1&{} 2 &{}1&{}1 \\ 2 &{} 3 &{} 1 &{}2 &{}2&{}2 \\ \end{array} \right)$$	k = 1		0.9562	0.0552	0.9441	0.0445
			k = 2	$\tau =7$	0.9348	0.0343	0.9659	0.0649
30	10	$$\left( \begin{array}{ccccc} 1&{} 2 &{} 1&{} 2&{} 1 \\ 3 &{} 2 &{} 2&{} 2&{} 4 \\ \end{array} \right)$$	k = 1		0.9628	0.0626	0.9630	0.0372
			k = 2	$\tau =6$	0.9712	0.0723	0.9272	0.0727
30	8	$$\left( \begin{array}{cccc} 1&{} 2 &{} 1 &{} 2 \\ 4 &{} 3 &{} 4 &{}5 \\ \end{array} \right)$$	k = 1		0.9290	0.0708	0.9714	0.0274
			k = 2	$\tau =5$	0.9801	0.0791	0.9207	0.0796
30	6	$$\left( \begin{array}{ccc} 2&{} 2 &{} 3 \\ 6 &{} 5 &{} 6 \\ \end{array} \right)$$	k = 1		0.9209	0.0203	0.9191	0.0797
			k = 2	$\tau =4$	0.9889	0.0896	0.9892	0.0112

**Table 2:** Frequentist coverage probabilities for ${\alpha }=0.95,0.05$ with true parameters ${\theta }=1,{\beta }=1$ and different hybrid censoring time

Censoring scheme				$Q_{\pi _{2}}({\theta })$		$Q_{\pi _{2}}({\beta })$	
n	m	$$\left( \begin{array}{cccc} R_{1}&{} R_{2} &{}\cdots \,R_{j-1}\\ R_{j} &{}0 \cdots 0 &{}R_{m} \\ \end{array} \right)$$		${\alpha }=0.95$	${\alpha }=0.05$	${\alpha }=0.95$	${\alpha }=0.05$
30	12	$$\left( \begin{array}{cccccc} 2&{} 1 &{} 1&{} 2 &{}1&{}1 \\ 2 &{} 3 &{} 1 &{}0 &{}0&{}4 \\ \end{array} \right)$$	$\eta$ = 10	0.9412	0.0587	0.9583	0.0579
		$$\left( \begin{array}{cccccc} 2&{} 1 &{} 1&{} 2 &{}1&{}1 \\ 2 &{} 0 &{} 0 &{}0 &{}0&{}6 \\ \end{array} \right)$$	$\eta$ = 8	0.9625	0.0373	0.9627	0.0372
		$$\left( \begin{array}{cccccc} 2&{} 1 &{} 1&{} 2 &{}1&{}0 \\ 0 &{} 0 &{} 0 &{}0 &{}0&{}9 \\ \end{array} \right)$$	$\eta$ = 6	0.9700	0.0297	0.9704	0.0708

Let ${\theta }^{\pi }({\alpha }|{\mathbf{data }})$ be the posterior ${\alpha }$-quantile of ${\theta }$ given ${\mathbf{data} }$. That is $F({\theta }^{\pi }({\alpha }|{\mathbf{data} })|{\mathbf{data} })={\alpha }$, here $F(\cdot |{\mathbf{data} })$ is the marginal posterior distribution of ${\theta }$. The frequentist coverage probability of this one side credible interval of ${\theta }$ is given by

$$\begin{aligned} Q^{\pi }({\alpha };{\theta })=P_{{\theta },{\beta }}(0<{\theta }\le {\theta }^{\pi }({\alpha }|{\mathbf{data} })). \end{aligned}$$

Similarly, Let ${\beta }^{\pi }({\alpha }|{\mathbf{data} })$ be the posterior ${\alpha }$-quantile of ${\beta }$ given ${\mathbf{data} }$. The frequentist coverage probability of this one side credible interval of ${\beta }$ is given by

$$\begin{aligned} Q^{\pi }({\alpha };{\beta })=P_{{\beta },{\theta }}(0<{\beta }\le {\beta }^{\pi }({\alpha }|{\mathbf{data} })). \end{aligned}$$

To sum up, take $Q^{\pi }({\alpha };{\theta })$ for an example, the computation of frequentist coverage probabilities are based on the following procedure.

1. Given the true value of ${\theta }$ and ${\beta }$, Typle-II APHCS samples ${\mathbf y }^{n}$ are generated from the distribution $Weibull({\theta },{\beta })$.
2. For each generated sample ${\mathbf y} ^{n}$, the posterior ${\alpha }$ quantile of ${\theta }$, ${\theta }^{\pi }({\alpha }|{\mathbf y }^{n})$, can be estimated by the above hybrid MCMC method.
3. Repeated *N* times for steps 1 and 2, the frequentist coverage probability $Q^{\pi }({\alpha };{\theta })$ can be estimated by the relative frequency
  $$\begin{aligned} \frac{\sharp \{{\theta }<{\theta }^{\pi }({\alpha }|{\mathbf y} ^{n})\}}{N}, \end{aligned}$$

where $\sharp \{{\theta }<{\theta }^{\pi }({\alpha }|{\mathbf y} ^{n})\}$ denotes the number of ${\theta }$ less than random variable ${\theta }^{\pi }({\alpha }|{\mathbf y} ^{n})$.

**Table 3:** Frequentist coverage probabilities for ${\alpha }=0.95,0.05$ with $n=30$, $R=(0,1,1,2,1,1,2,3,1,2,2,2)$ and different parameters true values

Parameter		$Q_{\pi _{2}}({\theta })$		$Q_{\pi _{2}}({\beta })$	
${\theta }$	${\beta }$	${\alpha }=0.95$	${\alpha }=0.05$	${\alpha }=0.95$	${\alpha }=0.05$
1	0.5	0.9528	0.0424	0.9457	0.0594
	1	0.9550	0.0591	0.9356	0.0577
	1.5	0.9460	0.0536	0.9527	0.0581
0.5	1	0.9432	0.0478	0.9539	0.0539
1.5		0.9458	0.0524	0.9578	0.0474

Tables 1, 2 and 3 can be obtained according to the above algorithm. Some of the points are quite clear from the numerical results.

- As expected, from Table 1, it is observed that the performances of all frequentist coverage probabilities become better when the sample size increases and censored sample size decreases, and they are sensitive to the stress levels *k*.
- The results are reported in Table 2 show that as the proportion of censored observations increase, the frequentist coverage probabilities decrease.
- However, as Table 3 presents that the frequentist coverage probabilities do not much sensitive to the parameter true values $({\theta },{\beta })$.

## Concluding remarks

Jeffreys prior, as one of the most important noninformative priors, is discussed under the SSPALT setting with Type-II adaptive progressive hybrid censored data. The likelihood function, which contains two cases of the adaptive progressive hybrid censoring data, is unified. Let $f(y|{\theta })\in \{f(y|{\theta }),y\in {\mathcal {Y}},{\theta }\in \Theta \}$, the Jeffreys priors for the survival models are obtained.

Taking Weibull distribution as an example, the Jeffreys priors based on the SSPALT with Type-II APHCS data are discussed, the other special cases also obtained. Besides, the posterior analyses based on these priors are studied. Employing Kullback–Leibler divergence as a measurement for the distance between two distributions, the permissibility of the priors is presented.

For one thing, given a prior, we can predict a future observation based on the observations by using the Bayesian predictive density function. However, there are few references study the prediction based on the noninformative priors. Work in these directions are currently under progress and we hope to report these findings in our future work. For another, note that an alternative generalisation of Kullback–Leibler divergence is ${\alpha }$-divergence suggested by Amari (1985), it will be of great interest to establish the permissibility for a prior based on the ${\alpha }$-divergence, more work may be needed along these directions.

## Appendix 1: Proof of Theorem 1

### *Proof*

From Eq. (4), we have

$$\begin{aligned} {\mathscr {L}}({\theta })& = {} \sum _{i\in D_{k}}\log f_{k}(y_{i}\mid {\theta })+\sum _{i\in C_{k}}\log S_{k}^{R_{i}}(y_{i}\mid {\theta })\\& = {} \sum _{i=1}^{m}{\mathscr {L}}_{i}^{D_{1}}({\theta }){\delta }_{1i}+\sum _{i=1}^{m}{\mathscr {L}}_{i}^{D_{2}}({\theta })\overline{{\delta }}_{1i} +\sum _{i=1}^{m}{\mathscr {L}}_{i}^{C_{1}}({\theta })R_{i}{\delta }_{1i}+\sum _{i=1}^{m}{\mathscr {L}}_{i}^{C_{2}}({\theta })R_{i}{\delta }_{2i}^{c}. \end{aligned}$$

Then

$$\begin{aligned} {\partial }^{2}_{hj}{\mathscr {L}}({\theta }) =\sum _{i=1}^{m}{\partial }^{2}_{hj}{\mathscr {L}}_{i}^{D_{1}}({\theta }){\delta }_{1i}+\sum _{i=1}^{m}{\partial }^{2}_{hj}{\mathscr {L}}_{i}^{D_{2}}({\theta })\overline{{\delta }}_{1i} +\sum _{i=1}^{m}{\partial }^{2}_{hj}{\mathscr {L}}_{i}^{C_{1}}({\theta })R_{i}{\delta }_{1i} +\sum _{i=1}^{m}{\partial }^{2}_{hj}{\mathscr {L}}_{i}^{C_{2}}({\theta })R_{i}{\delta }_{2i}^{c}. \end{aligned}$$

Notice that

$$\begin{aligned}&E_{{\theta }}\left[ \sum _{i=1}^{m}{\partial }^{2}_{hj}{\mathscr {L}}_{i}^{D_{1}}({\theta }){\delta }_{1i}\right] = \sum _{i=1}^{m}E_{{\theta }}\left[ {\partial }^{2}_{hj}{\mathscr {L}}_{i}^{D_{1}}({\theta }){\delta }_{1i}\right] \\&\quad = \sum _{i=1}^{m}E_{{\theta }}\left[ {\partial }^{2}_{hj}{\mathscr {L}}_{i}^{D_{1}}({\theta })\mid {\delta }_{1i} =1,{\theta }\right] P\left\{ {\delta }_{1i}=1\mid {\theta }\right\} ={\mathscr {E}}^{D_{1}}_{\sum }({\theta })P\left\{ {\delta }_{1i}=1\mid {\theta }\right\} . \end{aligned}$$

Similarly,

$$\begin{aligned}&E_{{\theta }}\left[ \sum _{i=1}^{m}{\partial }^{2}_{hj}{\mathscr {L}}_{i}^{D_{2}}({\theta })\overline{{\delta }}_{1i}\right] = {\mathscr {E}}^{D_{2}}_{\sum }({\theta })P\left\{ {\delta }_{1i}=0\mid {\theta }\right\} ,\\&\quad E_{{\theta }}\left[ \sum _{i=1}^{m}{\partial }^{2}_{hj}{\mathscr {L}}_{i}^{C_{1}}({\theta })R_{i}{\delta }_{1i}\right] ={\mathscr {E}}^{C_{1}}_{\sum }({\theta })P\left\{ {\delta }_{1i}=1\mid {\theta }\right\} ,\\&\quad E_{{\theta }}\left[ \sum _{i=1}^{m}{\partial }^{2}_{hj}{\mathscr {L}}_{i}^{C_{2}}({\theta })R_{i}{\delta }_{2i}^{c}\right] ={\mathscr {E}}^{C_{2}}_{\sum }({\theta })P\left\{ {\delta }_{2i}^{c}=1\mid {\theta }\right\} . \end{aligned}$$

Above equations we used the linearity of the mathematical expectation. Then, we get

$$\begin{aligned} J_{hj}& = {} -{\mathscr {E}}^{D_{1}}_{\sum }({\theta })P\left\{ {\delta }_{1i}=1\mid {\theta }\right\} - {\mathscr {E}}^{D_{2}}_{\sum }({\theta })P\left\{ {\delta }_{1i}=0\mid {\theta }\right\} \\&\quad - {\mathscr {E}}^{C_{1}}_{\sum }({\theta })P\left\{ {\delta }_{1i}=1\mid {\theta }\right\} -{\mathscr {E}}^{C_{2}}_{\sum }({\theta })P\left\{ {\delta }_{2i}^{c}=1\mid {\theta }\right\} \\& = {} -\left( H_{D}({\theta }_{h},{\theta }_{j})+H_{C}({\theta }_{h},{\theta }_{j})\right) . \end{aligned}$$

If the data are i.i.d., then the expectation does not depend on index *i*, we arrive at

$$\begin{aligned}&E_{{\theta }}\left[ \sum _{i=1}^{m}{\partial }^{2}_{hj}{\mathscr {L}}_{i}^{D_{1}}({\theta }){\delta }_{1i}\right] = \sum _{i=1}^{m}E_{{\theta }}\left[ {\partial }^{2}_{hj}{\mathscr {L}}_{i}^{D_{1}}({\theta }){\delta }_{1i}\right] \\&= \sum _{i=1}^{m}E_{{\theta }}\left[ {\partial }^{2}_{hj}{\mathscr {L}}_{i}^{D_{1}}({\theta })\mid {\delta }_{1i} =1,{\theta }\right] P\left\{ {\delta }_{1i}=1\mid {\theta }\right\} ={\mathscr {E}}^{D_{1}}({\theta })\sum _{i=1}^{m}P\left\{ {\delta }_{1i}=1\mid {\theta }\right\} . \end{aligned}$$

Similarly,

$$\begin{aligned}&E_{{\theta }}\left[ \sum _{i=1}^{m}{\partial }^{2}_{hj}{\mathscr {L}}_{i}^{D_{2}}({\theta })\overline{{\delta }}_{1i}\right] ={\mathscr {E}}^{D_{2}}({\theta })\sum _{i=1}^{m}P\left\{ {\delta }_{1i}=0\mid {\theta }\right\} , \\&\quad E_{{\theta }}\left[ \sum _{i=1}^{m}{\partial }^{2}_{hj}{\mathscr {L}}_{i}^{C_{1}}({\theta })R_{i}{\delta }_{1i}\right] = {\mathscr {E}}^{C_{1}}({\theta })\sum _{i=1}^{m}P\left\{ {\delta }_{1i}=1\mid {\theta }\right\} ,\\&\quad E_{{\theta }}\left[ \sum _{i=1}^{m}{\partial }^{2}_{hj}{\mathscr {L}}_{i}^{C_{2}}({\theta })R_{i}{\delta }_{2i}^{c}\right] = {\mathscr {E}}^{C_{2}}({\theta })\sum _{i=1}^{m}P\left\{ {\delta }_{2i}^{c}=1\mid {\theta }\right\} . \end{aligned}$$

$$\square$$

## Appendix 2: Proof of Theorem 2

### *Proof*

Notice that $n_{u}+n_{a}=m$, and

$$\begin{aligned} \sum _{i=1}^{m}P\left\{ {\delta }_{1i}=1\mid {\theta }\right\}& = {} E\left[ \sum _{i=1}^{m}{\delta }_{1i}\mid {\theta }\right] =E\left[ n_{u}\mid {\theta }\right] ,\\ \sum _{i=1}^{m}P\left\{ {\delta }_{1i}=0\mid {\theta }\right\}& = {} E\left[ \sum _{i=1}^{m} \overline{{\delta }}_{1i}\mid {\theta }\right] =E\left[ n_{a}\mid {\theta }\right] . \end{aligned}$$

If $$Y_{m}<\eta$$, then,

$$\begin{aligned} J_{hj}& = {} -{\mathscr {E}}^{D_{1}}_{\sum }({\theta })E\left[ n_{u}\mid {\theta }\right] - {\mathscr {E}}^{D_{2}}_{\sum }({\theta })E\left[ n_{a}\mid {\theta }\right] - {\mathscr {E}}^{C_{1}}_{\sum }({\theta })E\left[ c_{u}\mid {\theta }\right] - {\mathscr {E}}^{C_{2}}_{\sum }({\theta })E\left[ c_{a}\mid {\theta }\right] \\& = {} -{\mathscr {E}}^{D_{1}}_{\sum }({\theta })E\left[ n_{u}\mid {\theta }\right] - {\mathscr {E}}^{D_{2}}_{\sum }({\theta })E\left[ n_{a}\mid {\theta }\right] - {\mathscr {E}}^{C_{1}}_{\sum }({\theta })E\left[ n_{u}\mid {\theta }\right] - {\mathscr {E}}^{C_{2}}_{\sum }({\theta })E\left[ n_{a}\mid {\theta }\right] \\& = {} E\left[ n_{u}\mid {\theta }\right] \left( {\mathscr {E}}^{D_{2}}_{\sum }({\theta })+{\mathscr {E}}^{C_{2}}_{\sum }({\theta })- {\mathscr {E}}^{D_{1}}_{\sum }({\theta })-{\mathscr {E}}^{C_{1}}_{\sum }({\theta })\right) -m{\mathscr {E}}^{D_{2}}_{\sum }({\theta })-m{\mathscr {E}}^{C_{2}}_{\sum }({\theta }). \end{aligned}$$

If $Y_{j}<\eta <Y_{j+1}<Y_{m}$, then ${\delta }_{2i}^{c}={\delta }_{2i}^{c\eta }$, and $\sum _{i=1}^{m}{\delta }_{2i}^{c\eta }=j-n_{u}-1+1+1=j-n_{u}+1$, we have

$$\begin{aligned} J_{hj}& = {} -{\mathscr {E}}^{D_{1}}_{\sum }({\theta })E\left[ n_{u}\mid {\theta }\right] - {\mathscr {E}}^{D_{2}}_{\sum }({\theta })E\left[ n_{a}\mid {\theta }\right] - {\mathscr {E}}^{C_{1}}_{\sum }({\theta })E\left[ c_{u}\mid {\theta }\right] - {\mathscr {E}}^{C_{2}}_{\sum }({\theta })E\left[ c_{a}\mid {\theta }\right] \\& = {} -{\mathscr {E}}^{D_{1}}_{\sum }({\theta })E\left[ n_{u}\mid {\theta }\right] - {\mathscr {E}}^{D_{2}}_{\sum }({\theta })E\left[ n_{a}\mid {\theta }\right] - {\mathscr {E}}^{C_{1}}_{\sum }({\theta })E\left[ n_{u}\mid {\theta }\right] \\&\qquad - {\mathscr {E}}^{C_{2}}_{\sum }({\theta })E\left[ j-n_{u}+1\mid {\theta }\right] \\& = {} E\left[ n_{u}\mid {\theta }\right] \left( {\mathscr {E}}^{D_{2}}_{\sum }({\theta })+{\mathscr {E}}^{C_{2}}_{\sum }({\theta })- {\mathscr {E}}^{D_{1}}_{\sum }({\theta })-{\mathscr {E}}^{C_{1}}_{\sum }({\theta })\right) -m{\mathscr {E}}^{D_{2}}_{\sum }({\theta })-(j+1){\mathscr {E}}^{C_{2}}_{\sum }({\theta }). \end{aligned}$$

$$\square$$

## Appendix 3: Proof of Lemma 1

### *Proof*

Letting $$y_{ai}=\tau +k(y_{i}-\tau )$$$$(i=1,\dots ,m)$$. Substituting the density function (11) into Eq. (13), then take logarithm on both sides of the equation, we get

$$\begin{aligned}&\log L({\theta },{\beta };{\mathbf y} ^{m})\\&\quad =(n_{a}+c_{a})\log k+m\log {\beta }-m\log {\theta }+({\beta }-1)\sum _{i=1}^{n_{u}}\log \left( \frac{y_{i}}{{\theta }}\right) -\sum _{i=1}^{n_{u}}(1+R_{i})\left( \frac{y_{i}}{{\theta }}\right) ^{{\beta }} \\&\qquad +({\beta }-1)\sum _{i=n_{u}+1}^{j}\log \left( \frac{y_{ai}}{{\theta }}\right) -\sum _{i=n_{u}+1}^{j}(1+R_{i})\left( \frac{y_{ai}}{{\theta }}\right) ^{{\beta }} \\&\qquad +({\beta }-1)\sum _{i=j+1}^{m}\log \left( \frac{y_{ai}}{{\theta }}\right) -\sum _{i=j+1}^{m}\left( \frac{y_{ai}}{{\theta }}\right) ^{{\beta }}-R_{m}\left( \frac{y_{am}}{{\theta }}\right) ^{{\beta }}. \end{aligned}$$

Taking the partial derivatives with respect to ${\theta },{\beta }$, respectively, yields

$$\begin{aligned} {\mathcal {L}}_{20}& = {} \frac{{\partial }^{2}}{{\partial }{\theta }^{2}}\log L({\theta },{\beta };{\mathbf y} ^{m}) =\frac{m{\beta }}{{\theta }^{2}} -\sum _{i=1}^{n_{u}}\frac{(1+R_{i}){\beta }({\beta }+1)}{{\theta }^{2}}\left( \frac{y_{i}}{{\theta }}\right) ^{{\beta }} \\&-\sum _{i=n_{u}+1}^{j}\frac{(1+R_{i}){\beta }({\beta }+1)}{{\theta }^{2}}\left( \frac{y_{ai}}{{\theta }}\right) ^{{\beta }} -\sum _{i=j+1}^{m}\frac{{\beta }({\beta }+1)}{{\theta }^{2}}\left( \frac{y_{ai}}{{\theta }}\right) ^{{\beta }} -\frac{R_{m}{\beta }({\beta }+1)}{{\theta }^{2}}\left( \frac{y_{am}}{{\theta }}\right) ^{{\beta }}. \end{aligned}$$

Notice that the data are i.i.d., and $E(R_{1})={\mathcal {M}}$, combining (14) and (15) implies

$$\begin{aligned} J_{20}& = {} E_{({\theta },{\beta })}\left[ \frac{{\partial }^{2}}{{\partial }{\theta }^{2}}\log L({\theta },{\beta };{\mathbf y} ^{m})\right] =E_{({\theta },{\beta })}\left[ {\mathcal {L}}_{20}\right] \\& = {} \frac{m{\beta }}{{\theta }^{2}} -\sum _{i=1}^{n_{u}}\frac{(1+{\mathcal {M}}){\beta }({\beta }+1)}{{\theta }^{2}} -\sum _{i=n_{u}+1}^{j}\frac{(1+{\mathcal {M}}){\beta }({\beta }+1)}{{\theta }^{2}}{\mathscr {E}}^{1,0} \\&-\sum _{i=j+1}^{m}\frac{{\beta }({\beta }+1)}{{\theta }^{2}}{\mathscr {E}}^{1,0} -\frac{{\mathcal {M}}{\beta }({\beta }+1)}{{\theta }^{2}}{\mathscr {E}}^{1,0} \\& = {} \frac{m{\beta }}{{\theta }^{2}}-\frac{n_{u}(1+{\mathcal {M}}){\beta }({\beta }+1)}{{\theta }^{2}} -\frac{{\beta }({\beta }+1)}{{\theta }^{2}}\left[ \sum _{i=n_{u}+1}^{j}(1+{\mathcal {M}}){\mathscr {E}}^{1,0} +\sum _{i=j+1}^{m}{\mathscr {E}}^{1,0} +{\mathcal {M}}{\mathscr {E}}^{1,0}\right] \\& = {} \frac{m{\beta }}{{\theta }^{2}}-\frac{n_{u}(1+{\mathcal {M}}){\beta }({\beta }+1)}{{\theta }^{2}} -\frac{{\beta }({\beta }+1)}{{\theta }^{2}}\left( {\mathcal {M}}c_{a}+n_{a}\right) {\mathscr {E}}^{1,0} \\& = {} {\theta }^{-2}\left( m{\beta }-{\beta }({\beta }+1)(n_{u}({\mathcal {M}}+1)+ ({\mathcal {M}}c_{a}+n_{a}){\mathscr {E}}^{1,0})\right) . \end{aligned}$$

Similarly, we have

$$\begin{aligned} J_{02}& = {} {\beta }^{-2}\left( -m-n_{u}({\mathcal {M}}+1)({\gamma }_{2}+2{\gamma }_{1})- ({\mathcal {M}}c_{a}+n_{a}){\mathscr {E}}^{1,2}\right) ;\\ J_{11}& = {} {\theta }^{-1}\left( -m+n_{u}({\mathcal {M}}+1)(2+{\gamma }_{1})+ ({\mathcal {M}}c_{a}+n_{a})({\mathscr {E}}^{1,1}+{\mathscr {E}}^{1,0})\right) . \end{aligned}$$

$$\square$$

## Appendix 4: Proof of Theorem 9

### *Proof*

Observed that $f(y\mid {\theta })$ and $\pi ({\theta })$ are continuous on ${\mathcal {Y}}$ and $\Theta$, respectively, then the integral can be changed order under using the Fubini Theorem. Now, we check the first inequality.

18 $$\begin{aligned}&\idotsint \limits _{\prod _{i=1}^{n}{\mathcal {Y}}_{i}}{\mathcal {K}}\left\{ \pi ({\theta }\mid {\mathbf y} ^{n})\mid \pi _{0}({\theta }\mid {\mathbf y }^{n})\right\} m_{0}({\mathbf y} ^{n})d{\mathbf y} ^{n} \\&\quad =\idotsint \limits _{\prod _{i=1}^{n}{\mathcal {Y}}_{i}}\int _{\Theta _{0}}\log \left\{ \frac{\pi _{0}({\theta }\mid {\mathbf y} ^{n})}{\pi ({\theta }\mid {\mathbf y} ^{n})}\right\} \pi _{0}({\theta }\mid {\mathbf y} ^{n}) m_{0}({\mathbf y} ^{n})d{\theta }d{\mathbf y} ^{n} \\&\quad =\idotsint \limits _{\prod _{i=1}^{n}{\mathcal {Y}}_{i}}\int _{\Theta _{0}} \log \left\{ \frac{m({\mathbf y} ^{n})\pi _{0}({\theta })}{\pi ({\theta })m_{0}({\mathbf y} ^{n})} \right\} p({\mathbf y }^{n}\mid {\theta })\pi ({\theta })d{\theta }d{\mathbf y} ^{n} \\&\quad =\int _{\Theta _{0}}\log \left\{ \frac{\pi _{0}({\theta })}{\pi ({\theta })}\right\} \pi ({\theta })d{\theta }+\idotsint \limits _{\prod _{i=1}^{n}{\mathcal {Y}}_{i}} \log \left\{ \frac{m({\mathbf y} ^{n})}{m_{0}({\mathbf y} ^{n})} \right\} m_{0}({\mathbf y} ^{n}) d{\mathbf y} ^{n} \\&\quad \mathop {=}\limits ^{\mathrm {def}}I_{0}+I_{1}, \end{aligned}$$

where

19 $$\begin{aligned} I_{0}& = {} \int _{\Theta _{0}}\log \left\{ \frac{\pi _{0}({\theta })}{\pi ({\theta })}\right\} \pi ({\theta })d{\theta }. \\ I_{1}& = {} \idotsint \limits _{\prod _{i=1}^{n}{\mathcal {Y}}_{i}} \log \left\{ \frac{m({\mathbf y} ^{n})}{m_{0}({\mathbf y} ^{n})} \right\} m_{0}({\mathbf y} ^{n}) d{\mathbf y} ^{n} \\& = {} \idotsint \limits _{\prod _{i=1}^{n}{\mathcal {Y}}_{i}} \log \left\{ \frac{m({\mathbf y} ^{n-1}\mid y_{n})m(y_{n})}{m_{0}({\mathbf y} ^{n-1}\mid y_{n})m_{0}(y_{n})} \right\} m_{0}({\mathbf y} ^{n-1}\mid y_{n})m_{0}(y_{n}) d{\mathbf y} ^{n} \\& = {} \int _{{\mathcal {Y}}_{n}}\log \left\{ \frac{m(y_{n})}{m_{0}(y_{n})}\right\} m_{0}(y_{n})dy_{n}+ \idotsint \limits _{\prod _{i=1}^{n-1}{\mathcal {Y}}_{i}} \log \left\{ \frac{m({\mathbf y} ^{n-1}\mid y_{n})}{m_{0}({\mathbf y} ^{n-1}\mid y_{n})} \right\} m_{0}({\mathbf y} ^{n-1}\mid y_{n}) d{\mathbf y} ^{n-1} \\&\mathop {=}\limits ^{\mathrm {def}}&I_{11}+I_{12}. \end{aligned}$$

where

20 $$\begin{aligned} I_{11}& = {} \int _{{\mathcal {Y}}_{n}}\log \left\{ \frac{m(y_{n})}{m_{0}(y_{n})}\right\} m_{0}(y_{n})dy_{n}, \end{aligned}$$

21 $$\begin{aligned} I_{12}& = {} \idotsint \limits _{\prod _{i=1}^{n-1}{\mathcal {Y}}_{i}} \log \left\{ \frac{m({\mathbf y} ^{n-1}\mid y_{n})}{m_{0}({\mathbf y} ^{n-1}\mid y_{n})} \right\} m_{0}({\mathbf y} ^{n-1}\mid y_{n}) d{\mathbf y} ^{n-1}. \end{aligned}$$

Above equalities we used $\idotsint \limits _{\prod _{i=1}^{n-1}{\mathcal {Y}}_{i}} m_{0}({\mathbf y} ^{n-1}\mid y_{n}) d{\mathbf y} ^{n-1}=1$.

Notice that

$$\begin{aligned}&I_{0}+I_{12}\\& = {} \int _{\Theta _{0}}\log \left\{ \frac{\pi _{0}({\theta })}{\pi ({\theta })}\right\} \pi ({\theta })d{\theta }+\idotsint \limits _{\prod _{i=1}^{n-1}{\mathcal {Y}}_{i}} \log \left\{ \frac{m({\mathbf y} ^{n-1}\mid y_{n})}{m_{0}({\mathbf y} ^{n-1}\mid y_{n})} \right\} m_{0}({\mathbf y} ^{n-1}\mid y_{n}) d{\mathbf y} ^{n-1}\\& = {} \idotsint \limits _{\prod _{i=1}^{n-1}{\mathcal {Y}}_{i}}\int _{\Theta _{0}} \log \left\{ \frac{m({\mathbf y} ^{n-1}\mid y_{n})\pi _{0}({\theta })}{\pi ({\theta })m_{0}({\mathbf y} ^{n-1}\mid y_{n})} \right\} p({\mathbf y} ^{n-1}\mid y_{n}, {\theta })\pi ({\theta }))d{\theta }d{\mathbf y} ^{n-1}\\& = {} \idotsint \limits _{\prod _{i=1}^{n-1}{\mathcal {Y}}_{i}}\int _{\Theta _{0}} \log \left\{ \frac{\pi _{0}({\theta },y_{n}\mid {\mathbf y} ^{n-1})}{\pi ({\theta },y_{n}\mid {\mathbf y} ^{n-1})} \right\} p({\mathbf y} ^{n-1}\mid y_{n}, {\theta })\pi ({\theta }))d{\theta }d{\mathbf y} ^{n-1}\\& = {} \idotsint \limits _{\prod _{i=1}^{n}{\mathcal {Y}}_{i}}{\mathcal {K}}\left\{ \pi ({\theta },y_{n}\mid {\mathbf y} ^{n-1})\mid \pi _{0}({\theta },y_{n}\mid {\mathbf y} ^{n-1})\right\} m_{0}({\mathbf y} ^{n-1}\mid y_{n})d{\mathbf y} ^{n-1}. \end{aligned}$$

Using the concavity of $\log (t)$ on ${\mathbb {R}}^{+}$, we obtain

$$\begin{aligned} I_{11}=\int _{{\mathcal {Y}}_{n}}\log \left\{ \frac{m(y_{n})}{m_{0}(y_{n})}\right\} m_{0}(y_{n})dy_{n} \le \log \left\{ \int _{{\mathcal {Y}}_{n}}\frac{m(y_{n})}{m_{0}(y_{n})}m_{0}(y_{n})dy_{n}\right\} =0. \end{aligned}$$

Therefore, (18)–(21) imply that

$$\begin{aligned}&\idotsint \limits _{\prod _{i=1}^{n}{\mathcal {Y}}_{i}}{\mathcal {K}}\left\{ \pi ({\theta }\mid {\mathbf y} ^{n})\mid \pi _{0}({\theta }\mid {\mathbf y} ^{n})\right\} m_{0}({\mathbf y} ^{n})d{\mathbf y} ^{n} \\&\quad = \idotsint \limits _{\prod _{i=1}^{n}{\mathcal {Y}}_{i}}{\mathcal {K}}\left\{ \pi ({\theta },y_{n}\mid {\mathbf y} ^{n-1})\mid \pi _{0}({\theta },y_{n}\mid {\mathbf y} ^{n-1})\right\} m_{0}({\mathbf y} ^{n-1}\mid y_{n})d{\mathbf y} ^{n-1}\\&\qquad +\int _{{\mathcal {Y}}_{n}}\log \left\{ \frac{m(y_{n})}{m_{0}(y_{n})}\right\} m_{0}(y_{n})dy_{n}\\&\quad \le \idotsint \limits _{\prod _{i=1}^{n}{\mathcal {Y}}_{i}}{\mathcal {K}}\left\{ \pi ({\theta },y_{n}\mid {\mathbf y} ^{n-1})\mid \pi _{0}({\theta },y_{n}\mid {\mathbf y} ^{n-1})\right\} m_{0}({\mathbf y} ^{n-1}\mid y_{n})d{\mathbf y} ^{n-1}, \end{aligned}$$

that is the first inequality.

Similarly, for $k=2,3,\dots ,n$, we get

$$\begin{aligned}&\idotsint \limits _{\prod _{i=1}^{k}{\mathcal {Y}}_{i}}{\mathcal {K}}\left\{ \pi ({\theta },{\mathbf y} ^{k+1,n}\mid {\mathbf y} ^{k})\mid \pi _{0}({\theta },{\mathbf y} ^{k+1,n}\mid {\mathbf y} ^{k})\right\} m_{0}({\mathbf y} ^{k}\mid {\mathbf y} ^{k+1,n})d{\mathbf y} ^{k} \\&\quad = \idotsint \limits _{\prod _{i=1}^{k-1}{\mathcal {Y}}_{i}}{\mathcal {K}}\left\{ \pi ({\theta },{\mathbf y} ^{k,n}\mid {\mathbf y} ^{k-1})\mid \pi _{0}({\theta },{\mathbf y} ^{k,n}\mid {\mathbf y} ^{k-1})\right\} m_{0}({\mathbf y} ^{k-1}\mid {\mathbf y} ^{k,n})d{\mathbf y} ^{k-1}\\&\qquad +\int _{{\mathcal {Y}}_{k}}\log \left\{ \frac{m(y_{k}\mid {\mathbf y} ^{k+1,n})}{m_{0}(y_{k}\mid {\mathbf y} ^{k+1,n})}\right\} m_{0}(y_{k}\mid {\mathbf y} ^{k+1,n})dy_{k} \\&\quad \le \idotsint \limits _{\prod _{i=1}^{k-1}{\mathcal {Y}}_{i}}{\mathcal {K}}\left\{ \pi ({\theta },{\mathbf y} ^{k,n}\mid {\mathbf y} ^{k-1})\mid \pi _{0}({\theta },{\mathbf y} ^{k,n}\mid {\mathbf y} ^{k-1})\right\} m_{0}({\mathbf y} ^{k-1}\mid {\mathbf y} ^{k,n})d{\mathbf y} ^{k-1}. \end{aligned}$$

$$\square$$
